# Postoperative use of steroids for peri‐electrode edema after deep brain stimulation surgery: A retrospective cohort study

**DOI:** 10.1111/cns.14470

**Published:** 2023-09-16

**Authors:** Bin Wu, Yuting Ling, Changming Zhang, Jiakun Xu, Chao Yang, Nan Jiang, Ling Chen, Jinlong Liu

**Affiliations:** ^1^ Department of Neurosurgery, First Affiliated Hospital Sun Yat‐sen University Guangzhou China; ^2^ Department of Neurology, First Affiliated Hospital Sun Yat‐sen University Guangzhou China; ^3^ Department of Anesthesiology, First Affiliated Hospital Sun Yat‐sen University Guangzhou China

**Keywords:** deep brain stimulation, dexamethasone, Parkinson's disease, peri‐electrode edema, steroids

## Abstract

**Background:**

To review the incidence and extent of peri‐electrode edema after DBS and to clarify the effect of postoperative use of steroids on the peri‐electrode edema.

**Methods:**

This retrospective cohort study included 250 patients who underwent bilateral subthalamic nucleus (STN) DBS surgery with intact MRI within 1 month after DBS surgery. Patients were divided into steroid and non‐steroid groups, based on postoperative steroids use. The occurrence and extent of peri‐electrode edema were compared between the two groups, and other associated factors were analyzed using univariate and multivariate methods.

**Results:**

Peri‐electrode edema >1 cm^3^ in at least one hemisphere was reported in 215 (86.00%) patients. The mean volume of peri‐electrode edema observed in the steroid group was significantly smaller than in the non‐steroid group (8.09 ± 8.47 cm^3^ vs 17.10 ± 16.90 cm^3^, *p* < 0.001). In the steroid group, 104 (32.91%) of the 316 implanted electrodes present with edema less than 1 cm^3^, whereas in the non‐steroid group, only 27 (14.67%) of the 184 implanted electrodes present with edema less than 1 cm^3^ (*p* < 0.001). Multivariate analysis indicated that lesser peri‐electrode edema was significantly associated with postoperative steroids use and general anesthesia.

**Conclusions:**

Peri‐electrode edema is common after DBS surgery, and postoperative steroids use reduces the occurrence and extent of peri‐electrode edema.

## Graphical abstract

The postoperative use of steroids effectively reduced the extent of peri‐electrode edema after DBS surgery (steroid group vs non‐steroid group: 8.09 ± 8.47 cm^3^ vs. 17.10 ± 16.90 cm^3^, *p* < 0.001).

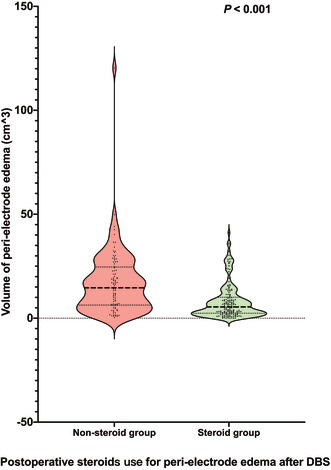

## 1 INTRODUCTION

Deep brain stimulation (DBS) surgery involves the implantation of electrodes into subcortical deep brain nucleus. Peri‐electrode edema is defined as edema of the brain tissue around the implanted electrodes, recognized by postoperative neuroimaging, which was not considered a common complication of DBS in previous studies. In a recent meta‐analysis study, the incidence of peri‐electrode edema was consumed as 35.8%, and only 8.7% of them were symptomatic.^1^ The patients with symptomatic peri‐electrode edema may present with headache, nausea, altered mental status, neurological deficits, seizures, and worsening of symptoms.^2^, ^3^, ^4^, ^5^, ^6^, ^7^, ^8^, ^9^ In most studies on peri‐electrode edema, neuroimaging was only performed in selected symptomatic patients, and the whole picture of peri‐electrode edema in all patients, based on routine neuroimaging scans, has seldom been reported. Furthermore, the risk factors and management of peri‐electrode edema after DBS surgery have been infrequently mentioned in previous studies. Steroids such as dexamethasone are commonly prescribed for the management of cerebral edema. However, the efficacy of the postoperative steroids in altering the development and natural course of peri‐electrode edema after DBS surgery is unknown.

In our center, 1.5‐T magnetic resonance imaging (MRI) was routinely performed on all patients who underwent DBS surgery, which was usually scheduled on the fifth postoperative day. At the initial stage, when we started to perform DBS surgery in 2006, we were relatively cautious about using steroids (dexamethasone) routinely to prevent postoperative cerebral edema due to a lack of experience, which has become routine in the perioperative management of DBS surgery for a long time. However, with increasing surgical experience and a lack of evidence, routine steroids prescribed after DBS surgery were abandoned in 2020. This transition offers an opportunity to explore the effect of steroids on the development of postoperative peri‐electrode edema. Therefore, this study included patients with Parkinson's disease (PD) who underwent subthalamic nucleus (STN) DBS surgery with postoperative MRI to review the incidence and extent of peri‐electrode edema. Furthermore, peri‐electrode edema was compared between patients with and without steroid prescription after surgery to determine whether steroids reduce the occurrence and extent of peri‐electrode edema. Other potential risk factors for peri‐electrode edema were also analyzed.

## 2 METHODS

### 2.1 Study design and data collection

**FIGURE 1:**
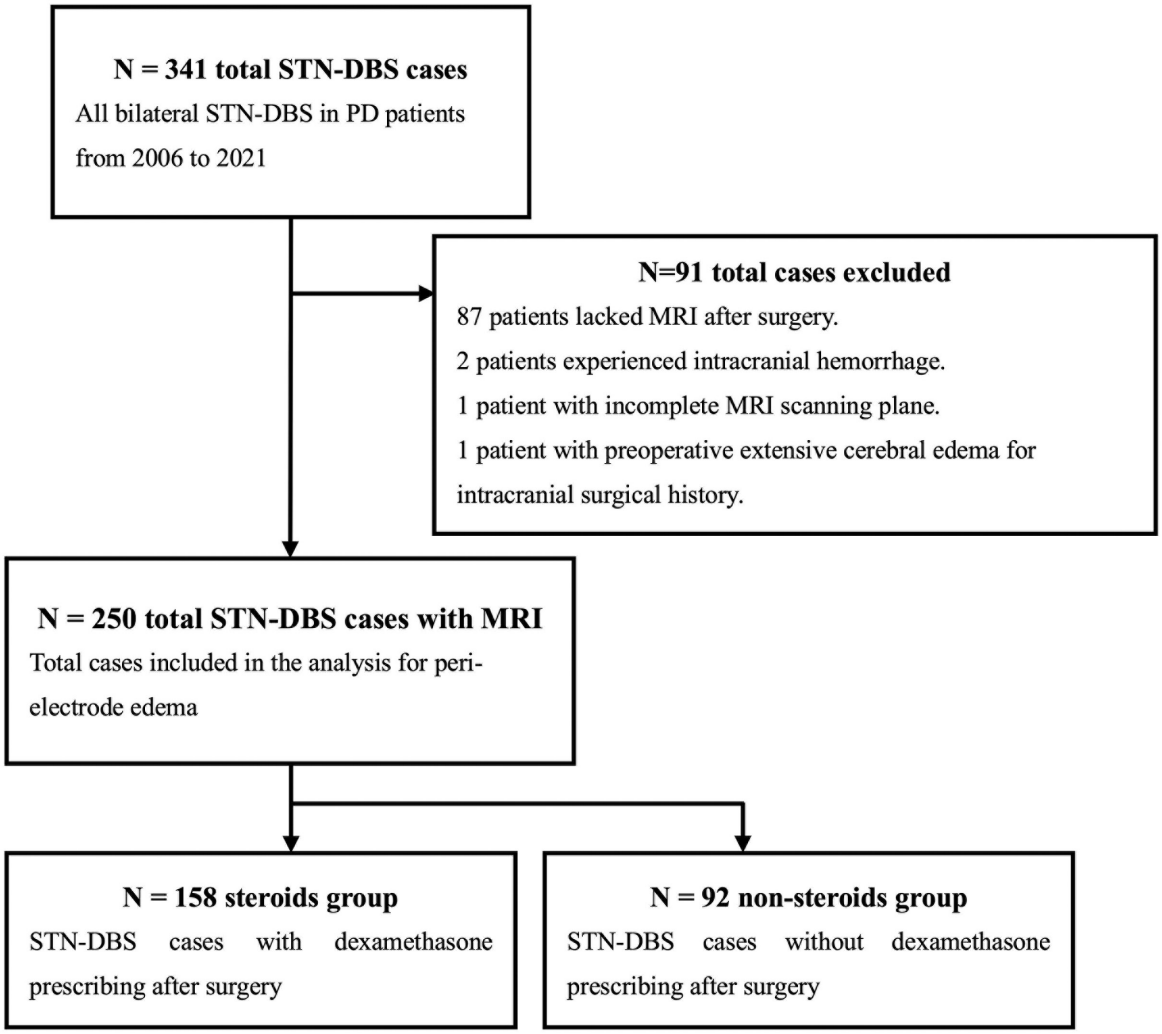
Patient population.

This retrospective cohort study initially identified 341 consecutive patients with PD who underwent bilateral STN‐DBS performed by a single neurosurgeon (Jinlong Liu) at the First Affiliated Hospital of Sun Yat‐sen University between 2006 and 2021. A total of 250 patients with intact MRI (1.5‐T) within one month after DBS surgery were included, and 91 patients were excluded (details listed in Figure 1). The clinical data of all patients, including population characteristics, intraoperative data, hospitalized clinical records, and postoperative neuroimaging, were collected. Based on the postoperative steroids use (dexamethasone), the patients were divided into steroid (*n* = 158) and non‐steroid (*n* = 92) groups (Figure 1). The occurrence and extent of peri‐electrode edema were compared between the two groups based on postoperative MRI findings, and other factors associated with peri‐electrode edema were also analyzed. The Ethics Committee for Clinical Research and Animal Trials of the First Affiliated Hospital of Sun Yat‐sen University approved this study (reference number: IIT2021‐142). The requirement for written informed consent was waived because of the retrospective analysis of routine clinical data and the absence of hazards and extra costs to patients.

### 2.2 DBS surgical procedure

Preoperative non‐stereotactic 3.0‐T MRI scans were performed in all patients. A T1‐weighted imaging scan was obtained to recognize whole‐brain anatomical structures, and a T2‐weighted imaging scan, or susceptibility‐weighted imaging (SWI) scan, was obtained to identify the STN. All anti‐Parkinsonian medications were withdrawn 12 h before DBS surgery. Each patient was positioned in a stereotactic frame (Leksell Coordinate Frame G; Elekta) under local anesthesia and computed tomography (CT) scanning was performed. Stereotactic CT images were merged with non‐stereotactic MRI images for surgical planning. Bilateral STNs were selected as targets for all patients, and trajectories were designed using a frontal approach to avoid the sulcus, vessels, and lateral ventricles. Surgeries were performed under general or local anesthesia during electrode implantation, and a single‐track microelectrode recording (MER) strategy was applied to identify the STN in all patients. Additional recording microelectrodes were inserted if required. After a satisfactory STN signal was obtained, a macroelectrode was implanted, followed immediately by a C‐arm scan to verify the electrode depth. Another electrode was implanted on the contralateral side following the same procedure, and an implantable pulse generator (IPG) was implanted under general anesthesia. Patients in the steroid group were administered intravenous dexamethasone (10 mg qd for 1 day within 24 h after surgery), followed by a stepwise decreased dose of oral dexamethasone tablets (1.5 mg q12 h for 3 days, then 1.5 mg qd for 3 days, and then 0.75 mg qd for 3 days). No postoperative steroids were prescribed to patients in the non‐steroid group.

### 2.3 Peri‐electrode edema measurement

**FIGURE 2:**
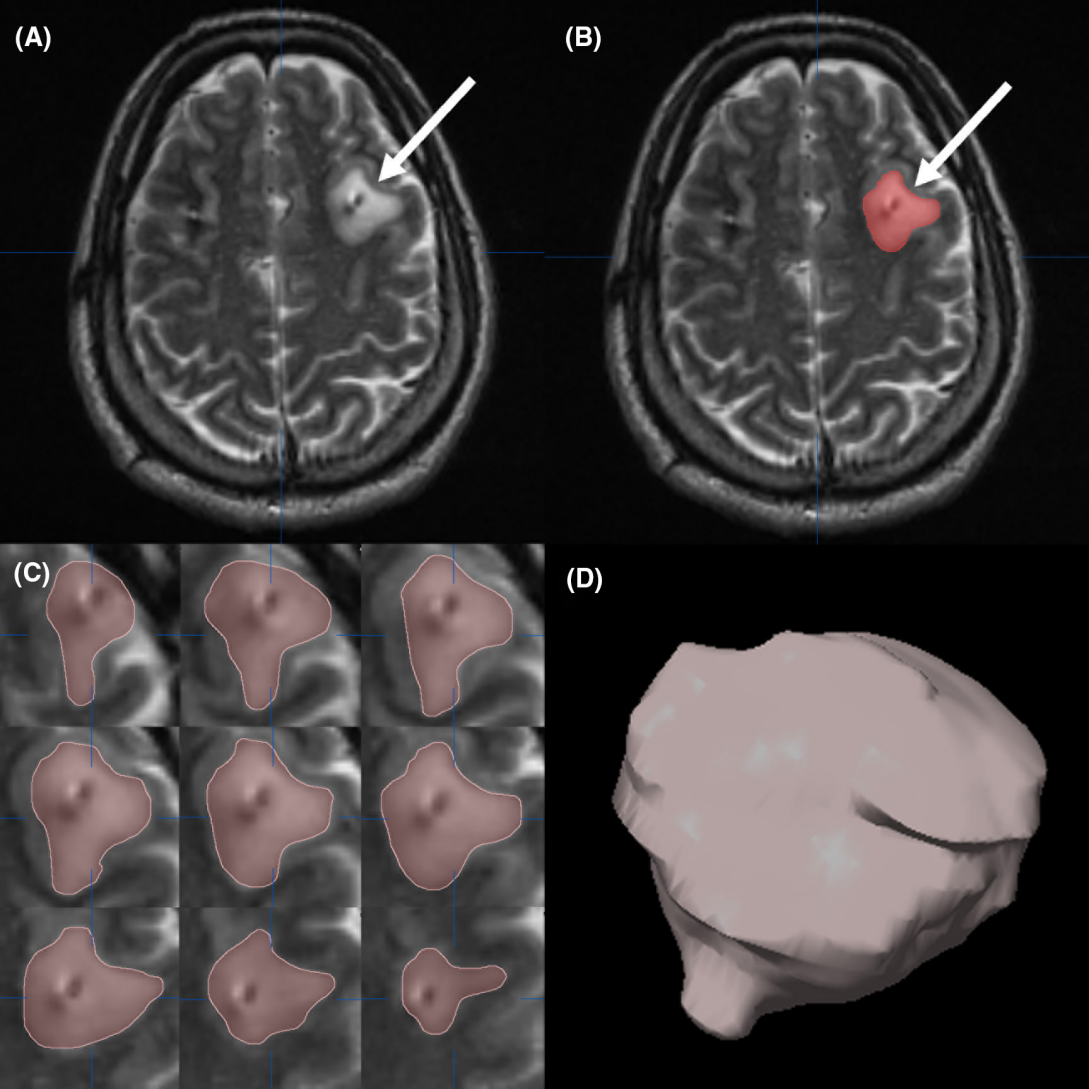
Measurement of volume of peri‐electrode edema. (A) Peri‐electrode edema on T2‐weighted MRI (the white arrow). (B) A mask covering the signal hyperintensity area was manually created on the slice (the white arrow). (C) The masks covering all signal hyperintensity areas alongside the electrode were segmented on each slice. (D) A three‐dimensional object representing the peri‐electrode edema was created based on the masks and volumetric estimation was performed using the software.

The measurement of peri‐electrode edema was based on postoperative 1.5‐T MRI, and all radiographic measurements were performed using the iPlanStereotax neuronavigation software (BrainLab) by an experienced neurosurgeon (Jiakun Xu) who was blinded to the patient groups. MRI scanning was usually scheduled on the fifth postoperative day; however, the MRI of some patients could not be scheduled on the fifth postoperative day for other reasons; thus, the study included all patients with MRI 1 month after surgery. Peri‐electrode edema was defined as signal hyperintensity surrounding the implanted electrodes on T2‐weighted MRI. In the object‐creation module of the software, a three‐dimensional mask covering all signal hyperintensity areas alongside the electrode was manually created on each slice. After the hyperintense area was segmented and a three‐dimensional object was created, volumetric estimation was performed using the software (Figure 2). For each patient, the volume of peri‐electrode edema was measured in both hemispheres, and the total volume of the left and right hemispheres was calculated and applied in the following analysis.

### 2.4 Statistical analysis

The descriptive analysis was performed to summarize the patients' baseline characteristics. Quantitative data were presented as means ± standard deviation (SD), and qualitative data were presented as the number of cases with percentages. An unpaired two‐tailed Student's *t*‐test was used to detect significant differences in continuous variables between the two groups, and an unpaired two‐tailed Welch's *t*‐test was used if the two populations had unequal variances. A Kolmogorov–Smirnov test was applied to assess data distribution, and an unpaired Mann–Whitney *U*‐test was applied if the parameters were not normally distributed. Pearson's chi‐squared test or Yates' correction for continuity was used to compare categorical variables. Pearson's correlation test was used to test the correlation between two continuous variables. Multivariate linear regression analysis was performed for risk factors associated with peri‐electrode edema. The threshold level of significance for all analyses was set at *p* < 0.05. Statistical analyses were performed using SPSS Statistics software (version 25.0; IBM Corp.).

## 3 RESULTS

### 3.1 Baseline Clinical characteristics

The study included 250 patients (152 men [60.80%] and 98 women [39.20%]) with a mean age of 59.86 ± 9.24 years and a mean PD disease duration of 9.84 ± 3.96 years. Table 1 summarizes the demographic and clinical characteristics of the patients in the steroid and non‐steroid groups.

**TABLE 1:** Comparison of characteristics between patients in steroid group and non‐steroid group.

	Steroid group (*n* = 158)	Non‐steroid group (*n* = 92)	*p* Value
Age (years)	58.53 ± 9.87	62.14 ± 7.56	0.005^a^ ^,^ *
PD duration (years)	9.91 ± 3.62	9.71 ± 4.51	0.222^a^
Sex			
Male	92 (58.23%)	60 (65.22%)	0.275^c^
Female	66 (41.77%)	32 (34.78%)	
Hypertension	29 (18.35%)	19 (20.65%)	0.656^c^
Diabetes mellitus	11 (6.96%)	8 (8.70%)	0.618^c^
Anesthesia methods			
Local anesthesia	2 (1.26%)	13 (14.13%)	<0.001^c^ ^,^ *
General anesthesia	156 (98.74%)	79 (85.87%)	
MER passage number	2.87 ± 1.23	2.26 ± 0.63	<0.001^a^ ^,^ *
Intraoperative MAP (mmHg)	78.59 ± 9.06	80.36 ± 6.71	0.079^b^
Surgery length (minutes)	350.76 ± 66.79	368.10 ± 64.06	0.030^a^ ^,^ *
Peri‐electrode edema volume			
MRI scanning time (d)	5.69 ± 3.19	5.08 ± 1.83	0.051^a^
Left (cm^3^)	3.71 ± 4.72	7.85 ± 6.88	<0.001^a^ ^,^ *
Right (cm^3^)	4.38 ± 5.60	9.25 ± 11.25	<0.001^a^ ^,^ *
Average (cm^3^)	8.09 ± 8.47	17.10 ± 15.90	<0.001^a^ ^,^ *
Postoperative seizures	4 (4.35%)	1 (0.63%)	0.120^d^

Abbreviations: MAP, mean arterial pressure; MER, microelectrode recordings; MRI, magnetic resonance imaging; PD, Parkinson's disease.

^a^ Unpaired Mann–Whitney *U*‐test.

^b^ Unpaired two‐tailed Welch's *t*‐test.

^c^ Pearson's chi‐squared test.

^d^ Yates's correction for continuity.

* Statistically significant at *P* < 0.05.

### 3.2 Postoperative steroids and peri‐electrode edema

Of the 250 patients, 215 (86.00%) had peri‐electrode edema >1 cm^3^ in at least one hemisphere (158 and 57 patients had bilateral and unilateral edema, respectively). Of the 500 implanted electrodes, 339 (67.80%) presented with peri‐electrode edema >1 cm^3^. The average volume of peri‐electrode edema for all patients was 11.41 ± 12.51 cm^3^ (range: 0–120.37 cm^3^). Steroid and non‐steroid groups included 158 patients (316 implanted electrodes) and 92 patients (184 implanted electrodes), respectively. The mean volume of peri‐electrode edema observed in the steroid group was significantly smaller than that observed in the non‐steroid group (8.09 ± 8.47 cm^3^ vs. 17.10 ± 16.90 cm^3^, *p* < 0.001, unpaired Mann–Whitney *U*‐test). In the steroid group, 104 (32.91%) of the 316 implanted electrodes presented with edema less than 1 cm^3^, whereas in the non‐steroid group, only 27 (14.67%) of the 184 implanted electrodes presented with edema less than 1 cm^3^. The difference was statistically significant (*p* < 0.001, Pearson's chi‐square test).

### 3.3 Postoperative MRI scanning time and peri‐electrode edema

**FIGURE 3:**
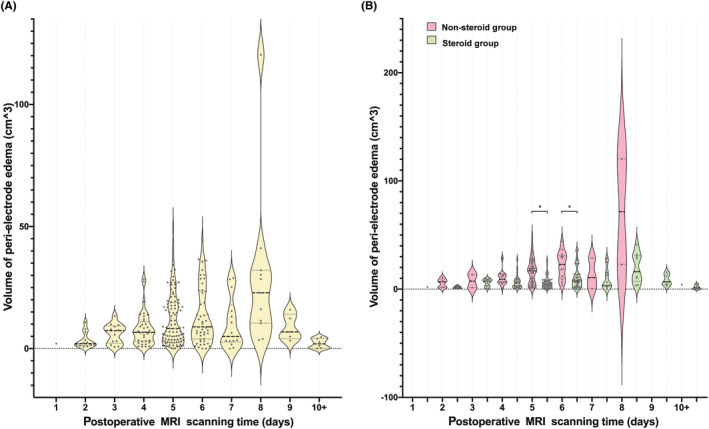
The relationship between peri‐electrode edema and postoperative MRI scanning time. (A) The average volume of peri‐electrode of each postoperative MRI scanning time for all patients (yellow violin plot). (B) The average volume of peri‐electrode of each postoperative MRI scanning time in non‐steroid group (red violin plot) and steroid group (green violin plot). * The average volume of peri‐electrode edema in the non‐steroid group was larger than that in the steroid group in day 5 and day 6 with statistically significant difference.

For all patients, the MRI scanning was performed an average of 5.46 ± 2.78 days after surgery. Figure 3A shows the relationship between postoperative MRI scanning time and peri‐electrode edema in all patients. Patients who underwent MRI on postoperative day 8 had the highest volume of peri‐electrode edema, with an average of 29.15 ± 32.62 cm^3^. Quadratic regression analysis indicated that peri‐electrode edema correlated with the timing of MRI scanning (*y* = −0.117*x* ^2^ + 3.002*x* − 0.618, *p* < 0.001). No difference in the average MRI scanning postoperative time (5.08 ± 1.83 days vs. 5.69 ± 3.19 days, *p* = 0.051, unpaired Mann‐Whitney *U*‐test) was observed between the groups. The average volume of peri‐electrode edema in non‐steroid group was larger than that in the steroid group in each postoperative time of MRI scanning; however, the difference was only statistically significant on day 5 (17.29 ± 11.85 cm^3^ vs. 6.66 ± 6.43 cm^3^, *p* < 0.001, unpaired Mann–Whitney *U*‐test) and day 6 (22.49 ± 12.46 cm^3^ vs. 10.39 ± 9.90 cm^3^, *p* = 0.002, unpaired Mann–Whitney *U*‐test) (Figure 3B). Similarly, the volume of peri‐electrode edema peaked on postoperative day 8 in both groups.

### 3.4 Other factors associated with peri‐electrode edema

Univariate and multivariate analyses were performed to identify the other factors associated with peri‐electrode edema. As mentioned above, peri‐electrode edema was associated with postoperative steroids use and postoperative MRI scanning time. Further univariate analysis indicated that peri‐electrode edema was associated with PD duration (*r* = −0.130, *p* = 0.040, Pearson's chi‐squared test) and anesthesia methods (local anesthesia vs. general anesthesia: 19.41 ± 14.99 cm vs. 10.90 ± 12.20 cm, *p* = 0.005, unpaired Mann–Whitney *U*‐test), but not associated with sex, age, history of hypertension or diabetes mellitus, MER passage number, intraoperative mean arterial pressure (MAP), or duration of surgery. Considering that the correlation between the volume of peri‐electrode edema and postoperative MRI scanning time was not linear but approximated to a quadratic function relation, we included patients who underwent MRI scanning on postoperative day 5 (*n* = 102, 49, and 53 from the steroid and non‐steroid groups, respectively) to perform further multivariable linear regression analysis. The multivariate model (stepwise method, *r* = 0.530, *F* = 19.351, *p* < 0.001, Durbin–Watson test value = 1.844) showed that peri‐electrode edema after DBS surgery was associated with postoperative steroids use (steroid vs. non‐steroid: *B* = −9.486, *t* = −4.943, *p* < 0.001) and anesthesia methods (local anesthesia vs. general anesthesia: *B* = 10.101, *t* = 2.479, *p* = 0.015).

## 4 DISCUSSION

In this case series, most patients developed peri‐electrode edema of different degrees after STN‐DBS surgery for PD based on postoperative MRI. The average volume of peri‐electrode edema reached its highest on the eighth postoperative day, which indicated that the peak of the peri‐electrode was approximately 1 week after surgery. Using a retrospective cohort design, we found that postoperative steroids use reduced the occurrence and extent of peri‐electrode edema. Further multivariate risk factor analysis confirmed these results and indicated that patients who underwent surgery under local anesthesia developed a larger extent of peri‐electrode edema.

In this study, peri‐electrode edema was not as rare as previously reported. The incidence of peri‐electrode edema >1 cm^3^ after DBS surgery was 86.00% for patients and 67.80% for electrodes, which was higher than that reported in most previous studies. More studies have reported only the incidence of symptomatic peri‐electrode edema, while neuroimaging scanning to recognize edema was only performed on selected patients who were symptomatic. However, the incidence of symptomatic peri‐electrode edema remains low. Kim et al.^10^ found that 7 (3.2%) of 221 patients with STN‐DBS displayed transiently increased low‐attenuation signals circumferentially surrounding the DBS electrodes and extending into the subcortical white matter on CT images. Fenoy et al.^3^ reported that 5 (3.4%) of 145 patients had peri‐lead and/or subcortical edema with new neurological symptoms based on CT scans after DBS surgery. Nazzaro et al.^4^ found that 10 (5.3%) of 189 patients with STN‐DBS presented with delayed symptomatic edema associated with a DBS lead based on CT scans. Sharma et al.^8^ reported that 16 (6.2%) of 260 patients with STN‐DBS (4.1% leads) developed symptomatic peri‐electrode edema on CT. Most patients with peri‐electrode edema were asymptomatic. Few studies have reported the total incidence of peri‐electrode involvement based on routine neuroimaging in the early postoperative stages. Ryu et al. reported increased subcortical signals on T2‐weighted MRI sequences along the DBS track in 15 (39%) of 38 patients.^11^ Englot et al.^6^ reported a low incidence of 10.5% (14 of 133 patients) for instances of T2 signal hyperintensity surrounding DBS leads on postoperative MRI scans (0–13 days postoperatively, average 5.1 ± 0.9 days). Saitoh et al.^12^ found that 6 (40.0%) of 15 patients showed peri‐electrode edema on MRI (3 to 10 days after surgery) without neurological deficits. Nolt et al.^13^ reported that 11 (84.6%) of 13 patients who underwent DBS exhibited white matter edema on at least one postoperative MRI, with none being symptomatic. Borellini et al.^7^ reported a higher incidence of 100% peri‐lead edema (7–20 days after surgery) in 15 patients based on a prospective MRI study. However, the sample sizes of these studies reported that the incidence of total peri‐electrode edema was relatively small, with only one study exceeding 100. Tian et al.^1^ performed a meta‐analysis and concluded that the incidence of peri‐lead edema was 35.8%, whereas that of symptomatic peri‐lead edema was 3.1%.

The incidence of peri‐electrodes after DBS surgery varies between different studies, and a higher incidence was reported in our case series, which could be attributed to the following three reasons. First, routine postoperative neuroimage scanning for all patients detected a higher incidence of peri‐electrode edema than those performed only on selected patients with symptoms, because most cases of peri‐electrode edema are asymptomatic. Second, MRI is more sensitive for detecting cerebral edema with a higher spatial resolution than CT, which improves the detection rate of postoperative peri‐electrode edema.^1^ MRI and CT have different sensitivities for detecting vasogenic edema in the presence of lead artifacts.^7^ Third, the timing of neuroimaging to detect peri‐electrode edema after DBS surgery is important. Peri‐electrode edema usually has a delayed onset; therefore, it is seldom detected on routine CT scans immediately after surgery.^3^, ^6^, ^7^, ^8^ In our study, patients who underwent MRI on postoperative day 8 had the highest volume of peri‐electrode edema. Nolt et al. evaluated the time course of white matter edema associated with DBS electrodes and found that maximal edema volumes occurred at postoperative week 2 in 8 (53.3%) of 15 electrodes.^13^ Englot et al.^6^ found that MRI scans with T2 signal abnormality were obtained significantly later after surgery (5.1 ± 0.9 days) than imaging without the finding (2.0 ± 0.1 days), and the occurrence of abnormal T2‐weighted MRI signal was more common on MRI obtained on or beyond the third postoperative day. As mentioned earlier, postoperative 1.5‐T MRI was routinely performed in all patients who underwent DBS surgery, which was usually scheduled on the fifth postoperative day within the increasing stage of edema. All signal hyperintensities surrounding the electrode on T2‐weighted MRI were considered indicative of peri‐electrode edema. Therefore, a high incidence of peri‐electrode edema was observed in the present study.

The risk factors for peri‐electrode involvement after DBS surgery have seldom been reported in previous studies. Whiting et al.^2^ reported that the occurrence of peri‐electrode edema was not associated with the presence of vascular disease, hypertension, anticoagulant or antiplatelet use, electrode target, index disease, unilateral versus bilateral lead placement, number of brain penetrations, or the presence or absence of MER. Englot et al.^6^ found no differences between patients with and without abnormal T2 signals, except for the time between surgery and MRI. Deogaonkar et al.^9^ reported no correlation between the number of MER tracts and the size or timing of the peri‐electrode edema. Nazzaro et al.^4^ reported a high incidence of symptomatic non‐hemorrhagic edema associated with the reimplantation of a previously removed lead. In our study, we found that the volume of peri‐electrode edema was associated with postoperative steroids use, timing of postoperative MRI, PD duration, and anesthesia methods but was not associated with sex, age, history of hypertension or diabetes mellitus, MER passage number, or surgery length in univariate analysis; only postoperative steroids usage and anesthesia methods remained statistically significant in multivariate analysis. Postoperative steroids use reduce the incidence and extent of peri‐electrode edema. Steroids can inhibit inflammatory responses, reduce microvascular permeability, stabilize cell membranes, restore sodium pump function, improve mitochondrial function, and prevent or attenuate free radical‐induced lipid peroxidation, all of which are effective in reducing edema.^1^ Interestingly, we found that patients under local anesthesia developed a larger volume of peri‐electrode edema after surgery than those under general anesthesia. Although the intraoperative blood pressure (MAP) was higher in patients under local anesthesia than general anesthesia (87.79 ± 6.97 mmHg vs. 78.70 ± 8.09 mmHg, *p* < 0.001, unpaired two‐tailed Student's *t*‐test), correlation between intraoperative MAP and peri‐electrode edema was not statistically significant. However, intraoperative blood pressure is more unstable in patients under local anesthesia during MER and macroelectrode implantation, which may cause more damage to the brain tissue around the trajectories. Meanwhile, the small sample size of patients under local anesthesia may introduce bias into the results. Further research is required to clarify the impact of anesthesia methods on peri‐electrode edema.

The peri‐electrode edema may be the result of an impaired blood brain barrier (BBB) with increased permeability.^9^, ^10^, ^14^ During electrode implantation in surgery, local microhemorrhage and mechanical trauma are caused by microelectrodes or macroelectrodes passing through the brain tissue. Other potential reasons that may cause peri‐electrode edema include the flow of cerebrospinal fluid along the electrode trajectory, cerebral venous infarction, immune hypersensitivity reactions, neurotoxicity or inflammatory reactions caused by implanted materials, infection, and hemorrhage.^1^

Steroids are commonly reported to manage symptomatic peri‐electrode edema after DBS surgery,^2^, ^3^, ^4^, ^5^, ^6^, ^9^, ^10^, ^15^ while another study recommended that no corticosteroid treatment should be administered to patients whose MRI shows peri‐lead edema in the first 7–60 days after surgery because of concerns about overtreatment and iatrogenic complications.^7^ However, no previous studies have reported the prophylactic methods for peri‐electrode edema. At the initial stage in 2006, when we started to perform DBS surgery at our center, we were relatively cautious in using steroids routinely to prevent postoperative cerebral edema, which became a clinical routine in the following years for the perioperative management of DBS surgery until 2020. Because of the increasing surgical experience and lack of evidence to support the use of steroids for edema after DBS surgery, routine postoperative steroids were abandoned. This transition offered an opportunity to explore the effect of steroids on the development of postoperative peri‐electrode edema, which led to this retrospective cohort study. Spataro et al. reported that a 6‐day treatment with dexamethasone after device insertion in a rat neocortex could control reactive responses around the inserted neural prosthetic devices by attenuating astroglial responses and might provide a means to ensure their long‐term function.^16^ In this study, we found that the postoperative administration of dexamethasone reduced the occurrence and extent of peri‐electrode edema, and no apparent adverse effects from steroids use were observed. Most cases of peri‐electrode edema in this study were asymptomatic. Previous studies have indicated that patients with simple asymptomatic peri‐electrode edema can recover without specific treatment, with a recovery time ranging from 1 to 70 days, with a mean of 31.6 days.^1^ Postoperative seizures were more frequent in the non‐steroid group than in the steroid group (4.35% vs. 0.63%), which might be indirectly caused by the extended peri‐electrode edema, and the difference did not reach statistical significance for the low incidence of seizures. However, data concerning other long‐term outcomes were not available for comparison between the two groups in this study. Therefore, the study findings are insufficient to support the postoperative use of steroids for peri‐electrode edema, and future prospective studies with a higher level of evidence are warranted.

To date, this study is the first to report the occurrence and extent of peri‐electrode edema after DBS surgery in a large sample based on routine postoperative MRI. To our knowledge, this is the first study to report the effect of postoperative steroids use on the development of peri‐electrode edema after DBS surgery. However, this study had some limitations. First, the retrospective nature of the study might have introduced bias in the group comparison analysis. For example, the mean age of the two groups differed, although further univariate and multivariate analyses indicated that age was not associated with the peri‐electrode. Second, owing to the retrospective nature of the study, we were unable to confirm the correlation between new‐onset symptoms and edema; therefore, the incidence of symptomatic edema was not available. Third, long‐term clinical outcomes were not compared between the two groups, which requires further prospective studies.

## 5 CONCLUSION

Peri‐electrode edema is a common complication of DBS surgery and reaches a peak at approximately 1 week postoperatively, based on the observations of this study. Postoperative steroids use reduces the occurrence and extent of peri‐electrode edema. However, further prospective studies are needed to determine whether steroids should be used to prevent peri‐electrode edema after deep brain stimulation surgery.

## AUTHOR CONTRIBUTIONS

Bin Wu performed the data analyses and wrote the manuscript; Jinlong Liu and Bin wu designed the study; Yuting ling and Changming Zhang collected the study data; Jiakun Xu performed the radiological measurement; Jinlong Liu, Ling Chen, Nan Jiang, and Chao Yang revised the manuscript. All authors read and approved the final version of the manuscript.

## FUNDING INFORMATION

This study was funded by grants from the National Natural Science Foundation of China (Nos. 82271267 and 81902527) and Guangdong Provincial Scientific Key R&D Program (number 2018B030337001).

## CONFLICT OF INTEREST STATEMENT

The authors declare that they have no conflict of interest.

## Data Availability

The data that support the findings of the study are available from the corresponding author upon reasonable request.
